# Thermoelectric Properties of Co-Substituted Al–Pd–Re Icosahedral Quasicrystals

**DOI:** 10.3390/ma15196816

**Published:** 2022-09-30

**Authors:** Yoshiki Takagiwa

**Affiliations:** National Institute for Materials Science (NIMS), 1-2-1 Sengen, Tsukuba 305-0047, Japan; takagiwa.yoshiki@nims.go.jp

**Keywords:** quasicrystals, thermoelectric materials, Al–Pd–Re–Co, electron doping

## Abstract

The practical application of quasicrystals (QCs) as thermoelectric materials makes icosahedral (*i*-) Al–Pd–Re QC attractive because of its moderate electrical conductivity (~280 Ω^−1^ cm^−1^), relatively high Seebeck coefficient (~100 μV K^−1^), and low thermal conductivity (~1.3 W m^−1^ K^−1^) at room temperature. To develop a thermoelectric Π-shaped power generation module, we need both p- and n-type thermoelectric materials. In this work, we aimed to develop an n-type *i*-Al–Pd–Re-based QC and investigated the effect of Co substitution for Re on the thermoelectric properties, i.e., the electron-doping effect. We synthesized dense bulk samples with nominal compositions of Al_71_Pd_20_(Re_1−*x*_Co*_x_*)_9_ (*x* = 0, 0.1, 0.2, 0.3, 0.4, 0.5) via arc-melting, annealing, and sintering methods. We found that Co can produce n-type carriers in dilute substitution amounts of *x* = 0.1 and 0.2; however, the Seebeck coefficient at 300 K showed an n- to p-type transition with increasing *x*. This indicates that a simple rigid-band approximation is not applicable for *i*-Al–Pd–Re QC, which makes it difficult to synthesize an n-type *i*-Al–Pd–Re-based QC. Although the thermal conductivity was reduced from 1.28 (*x* = 0) to 1.08 W m^−1^ K^−1^ (*x* = 0.3) at 373 K by lowering of the electron thermal conductivity (electrical conductivity) and the alloying effect via Co substitution, the dimensionless figure of merit was not enhanced because of lowering of the power factor for all samples. The elastic moduli of *i*-Al–Pd–Re QC decreased by Co substitution, indicating that *i*-Al–Pd–Re-Co QC had a more ionic and brittle character.

## 1. Introduction

From the viewpoint of highly efficient energy use, the importance of thermoelectric conversion technology that directly converts thermal energy into electrical energy is increasing. There are many reports on research and development related to various high-performance thermoelectric materials [1] and modularization technology [2]. The dimensionless figure of merit (*zT*) is an evaluation index for a thermoelectric material, expressed as:

(1) $$zT=\frac{S^{2}\sigma }{\kappa_{\mathrm{total}}}T,$$

where *S*, *σ*, *κ*_total_, and *T* are the Seebeck coefficient, electrical conductivity, total thermal conductivity, and temperature, respectively [3,4]. Here, *κ*_total_ is the sum of two contributions: the electron contribution, *κ*_electron_, and the phonon contribution, *κ*_phonon_, i.e.,

(2) $$\kappa_{\mathrm{total}}=\kappa_{\mathrm{electron}}+\kappa_{\mathrm{phonon}}$$

*κ*_electron_ is proportional to *σ* through the Wiedemann–Franz law:

(3) $$\kappa_{\mathrm{electron}}=L_{0}\sigma T,$$

where *L*_0_ is the Lorenz number. Thus, both optimizing *S* and *σ* and lowering *κ*_phonon_ are necessary to enhance the *zT* value. Relevant materials designs, such as band engineering and valleytronics [5], can improve the power factor (*S*^2^*σ*), and nano-structuring [6] and phonon engineering [2] can be employed to reduce *κ*_phonon_.

In recent years, the application of thermoelectric materials as autonomous power supplies to drive Internet-of-Things (IoT) devices [7] has become possible [8,9]. The usable temperature range is below 200 °C, as derived from environmental heat sources and low-temperature waste heat, and it is crucial to ensure a temperature difference when using thermoelectric power generation modules. For this purpose, the *κ*_total_ of a material-forming thermoelectric module should be low, i.e., less than a few W m^−1^ K^−1^ at operating temperature. In contrast, a high output voltage is required when driving IoT devices with a DC–DC converter; thus, a high *S* is required for the material.

Icosahedral quasicrystals (*i*-QCs) exhibit interesting electrical and thermal transport properties [10], and their applications [11] vary in structural materials [12], thermal rectifiers [13], and thermoelectric materials [14]. Regarding thermoelectric applications, *i*-Al–Pd–Re QC has been widely investigated from experimental [14] and theoretical [15] points of view. The *i*-Al–Pd–Re QC exhibits promising thermoelectric properties because of its moderate *σ* (~280 Ω^−1^ cm^−1^), relatively high *S* (~100 μV K^−1^), and low *κ*_total_ (~1.3 W m^−1^ K^−1^) at room temperature [16], which are brought about by pseudo-gap formation at the Fermi energy (*E*_F_) [17] and their complex crystal structures with icosahedral symmetry [18]. Recently, we obtained a relatively large *zT*_max_ value of 0.26 at 573 K for the Fe-substituted Al–Pd–Re system [16]: 2/1-Al–Pd–Re–Fe approximant crystal showed the highest *S*^2^*σ* of ~900 μW m^−1^ K^−2^ at 573 K. To date, we have succeeded in improving the thermoelectric performance of only p-type materials based on *i*-Al–Pd–Re QC [14,16] and isostructural *i*-Al–Pd–Mn QC [19,20].

To develop a robust thermoelectric Π-shaped power generation module, we need both p- and n-type thermoelectric materials with good mechanical properties. In this work, we aimed to develop an n-type *i*-Al–Pd–Re-based QC. We selected Co as an electron dopant because Co has a larger number of valence electrons than Re. Regarding the Al–Pd–Co ternary system, there is one report on the thermoelectric properties of 1/1-cubic approximant crystal [21]. This paper reports the effect of Co substitution for Re in *i*-Al–Pd–Re QC on the thermoelectric and mechanical properties.

## 2. Methods and Materials

Mother ingots of nominal compositions of Al_71_Pd_20_(Re_1−*x*_Co*_x_*)_9_ (*x* = 0, 0.1, 0.2, 0.3, 0.4, 0.5) were synthesized by an arc-melting technique under a purified argon atmosphere (NEV-AD03TC; Nisshin Giken Co., Japan) using the starting materials Al (powder, 99.99%), Pd (powder, 99.9%), Re (powder, 99.9%), and Co (powder, >99%). The obtained bulk samples were annealed at 1223 K for 24 h under a purified argon atmosphere to prevent oxidization (MILA-5000; Advance Riko, Inc., Yokohama, Japan). The annealed samples were hand-milled in ethanol using an agate mortar and pestle. The hand-milled powder samples were sieved using a stainless-steel sieve of 45 μm mesh and then placed in a carbon die with an inner diameter of 10 mm for spark plasma sintering (SPS) (LABOX-110MC; SinterLand, Inc., Nagaoka, Japan) under a purified argon atmosphere. The applied pressure was set to 115 MPa during the sintering process to synthesize dense bulk samples. This value is twice as high as a condition that previously achieved a high relative density above 90% for *i*-Al–Pd–Re QC [22]. To obtain dense bulk samples, we performed 10 min at a sintering temperature (*T*_S_) of 1223–1233 K, at which the degree of shrinkage was saturated. Table 1 lists *T*_S_ and bulk densities (*d*_bulk_) obtained from the Archimedes method for Al_71_Pd_20_(Re_1−*x*_Co*_x_*)_9_ (*x* = 0, 0.1, 0.2, 0.3, 0.4, 0.5). Here, the data for the sample with *x* = 0 are quoted from a reported paper [16].

Phase characterization of the samples was evaluated by X-ray diffraction (XRD) with Cu *K*α radiation (Mini-Flex 600; Rigaku, Inc., Akishima, Japan) and a scanning thermal probe micro-imaging apparatus (STPM-1000; Advance-Riko, Inc., Yokohama, Japan). The *σ* and *S* values were measured between 300 and 873 K by the four-probe and steady-state temperature gradient methods, respectively (ZEM-3; Advance Riko, Inc., Yokohama, Japan). The *κ*_total_ value was calculated from *d*_bulk_, the specific heat at constant pressure (*C*_P_), and thermal diffusivity (*λ*) using the relationship *κ*_total_ = *d*_bulk_∙*C*_P_∙*λ*. *C*_P_ was measured using differential scanning calorimetry (DSC404-F3; NETZSCH Japan, Yokohama, Japan) and *λ* was measured by a light flash apparatus (LFA467-HT; NETZSCH Japan, Yokohama, Japan) from 373 to 873 K. The longitudinal (*v*_long_) and transverse (*v*_trans_) speeds of sound were measured by the ultrasonic pulse-echo method (Echometer 1062; Nihon Matech Corp., Tokyo, Japan). For rough estimations of the elastic moduli (Poisson’s ratio (*ν*), Young’s modulus (*E*), shear modulus (*G*), and bulk modulus (*B*)) for these QC samples, the following relations for isotropic materials were used for the calculations [23,24,25]:

(4) $$\nu =\frac{v_{\mathrm{long}}^{2}-2v_{\mathrm{trans}}^{2}}{2\left(v_{\mathrm{long}}^{2}-v_{\mathrm{trans}}^{2}\right)},$$

(5) $$E=\frac{d_{\mathrm{bulk}}v_{\mathrm{long}}^{2}\left(3v_{\mathrm{long}}^{2}-4v_{\mathrm{trans}}^{2}\right)}{v_{l}^{2}-v_{t}^{2}},$$

(6) $$G=d_{\mathrm{bulk}}v_{\mathrm{trans}}^{2},$$

(7) $$B=d_{\mathrm{bulk}}\left(v_{\mathrm{long}}^{2}-\frac{4}{3}v_{\mathrm{trans}}^{2}\right).$$

## 3. Results and Discussion

The relative density of the sample with *x* = 0 was 95.7% [16], indicating that a dense bulk sample was fabricated by the SPS process. The ideal densities of the Co-substituted samples were calculated from *d*_bulk_ (relative density: ~100%, 6.30 g cm^–3^) of the sample with *x* = 0 [22] and the rate of change in average atomic weight (Table 1). As a result, it was found that the dense bulk samples with relative densities of >95% were fabricated for Co-substituted samples. The overall trend of *d*_bulk_ decreased with increasing Co concentration *x*, which can be understood as decreasing the average mass by substitution of the lighter element Co for Re.

**Figure 1:**
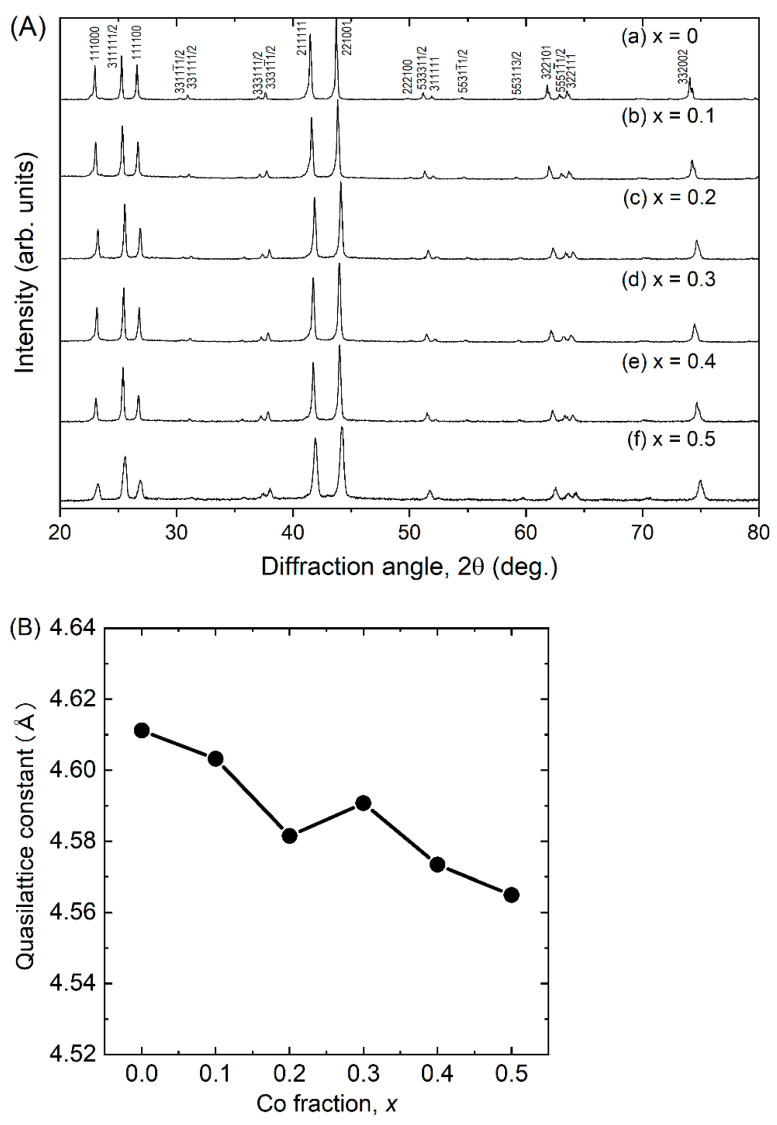
(**A**) X-ray diffraction patterns and (**B**) quasi-lattice constant of Al_71_Pd_20_(Re_1−*x*_Co*_x_*)_9_ (*x* = 0 [16], 0.1, 0.2, 0.3, 0.4, 0.5).

We confirmed that all synthesized bulk samples were identified as an *F*-type quasi-crystalline phase [26], as shown in Figure 1A. In the Al–Pd–Re–Co system, the formation of 2/1- or 1/1-approximant crystal was not realized, unlike in Al–Pd–Re–Ru [27] and Al–Pd–Re–Fe [16] quaternary systems. We observed peak shifting to a higher degree with increasing *x*, indicating that the quasi-lattice constant (*a*_R_) decreased by Co substitution for Re (Figure 1B). This trend can be qualitatively explained by substitution of the smaller atomic radii of Co (0.125 nm) for Re (0.138 nm). However, we observed that the sample with *x* = 0.3 had a slight increase in *a*_R_, which may be brought about by a composition deviation, as discussed below.

**Figure 2:**
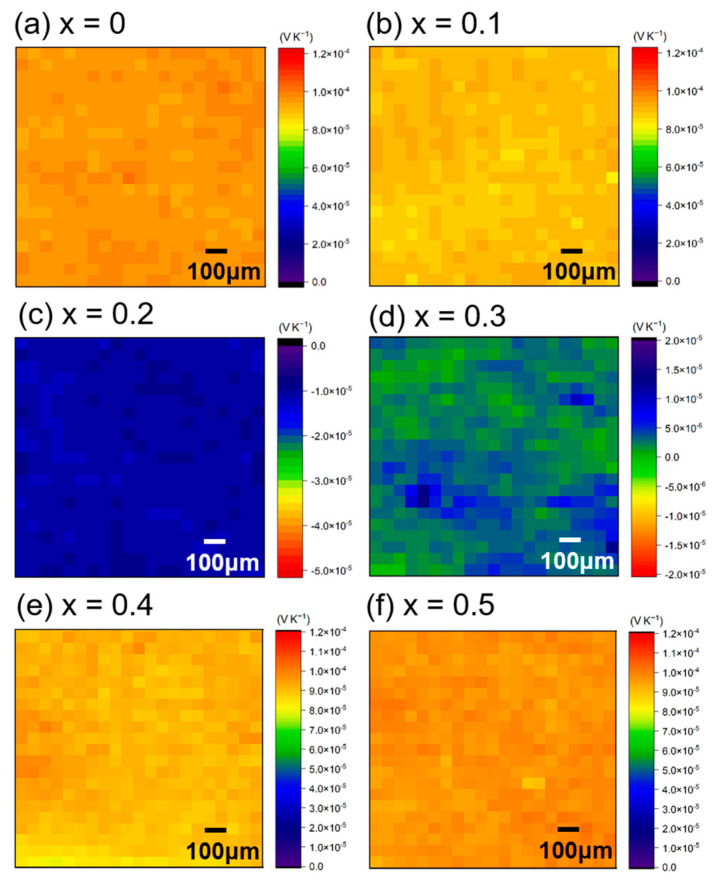
Seebeck coefficient mapping measurements at room temperature for Al_71_Pd_20_(Re_1−*x*_Co*_x_*)_9_ (*x* = 0 [16], 0.1, 0.2, 0.3, 0.4, 0.5).

The morphology of the dense bulk samples synthesized by the SPS process did not change with varying *x*. To assess the sample’s quality, the mapping measurements of *S* at 300 K, which is sensitive to local composition, were performed (Figure 2). As a result, homogeneous microstructures were obtained, except for the sample with *x* = 0.3, and there was no secondary precipitation. These results agree with the XRD measurements. The sample with *x* = 0.3 had a composite microstructure consisting of p- and n-type compositions with small absolute values of *S*. When *x* increased, the carrier type changed from p- to n-type, then turned to p-type again. However, its magnitude of *S* for an n-type material is relatively low (−10 μV K^−1^) for the sample with *x* = 0.2.

**Figure 3:**
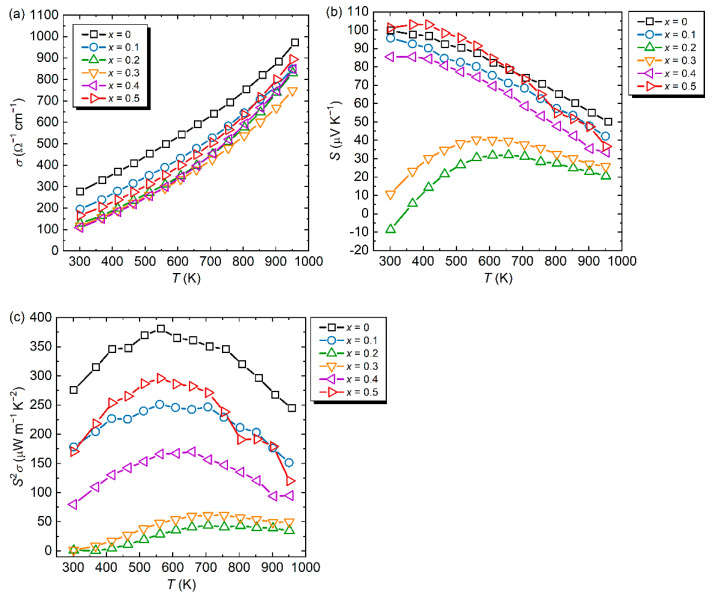
(**a**) Electrical conductivity (*σ*), (**b**) Seebeck coefficient (*S*), and (**c**) power factor (*S*^2^*σ*) as functions of temperature for Al_71_Pd_20_(Re_1−*x*_Co*_x_*)_9_ (*x* = 0 [16], 0.1, 0.2, 0.3, 0.4, 0.5).

Figure 3a–c show the temperature dependence of *σ*, *S*, and *S*^2^*σ* from 300 to 873 K for the Al_71_Pd_20_(Re_1−*x*_Co*_x_*)_9_ (*x* = 0 [16], 0.1, 0.2, 0.3, 0.4, 0.5) samples. All samples had a similar positive temperature coefficient of *σ* (semiconducting behavior), with a varying magnitude of 100–280 Ω^−1^ cm^−1^ at 300 K. The calculated activation energy from the Arrhenius plot was 0.11 eV for the sample with *x* = 0 and increased to 0.15 eV with Co substitution. Compared with transition metal-substituted *i*-Al–Pd–Re QCs, these values are comparable with those of *i*-Al–Pd–Re–Ru QC (0.05–0.12 eV) [28] and *i*-Al–Pd–Re–Fe QC (0.14–0.17 eV) [16]. Since *S* at 300 K decreased owing to Co substitution for Re, the decrease in carrier concentration was not the cause of the decrease in *σ*, i.e., lowering of the relaxation time owing to chemical disordering of Co at Fe sites. The absolute value of *S* for p-type materials (except for samples with *x* = 0.2 and 0.3) decreased with the increasing temperature, which is attributed to an increase in minority carriers. This is consistent with the temperature dependence of *σ*. The *S*^2^*σ* value showed a similar temperature dependence, with a peak at mid-temperatures of 600–700 K for all samples, and exhibited a maximum value of ~380 μW m^−1^ K^−2^ at 573 K for the unsubstituted sample, i.e., *S*^2^*σ* was not improved by Co substitution for Re because of the reduction of *S* and *σ*.

The highest value of *σ* at 300 K was measured for the *i*-Al_71_Pd_20_Re_9_ QC (~280 Ω^−1^ cm^−1^) [16]: *σ* decreased up to *x* = 0.4 and then increased for the sample with *x* = 0.5. This non-monotonic change in *σ* with varying *x* cannot be understood only in terms of the change in carrier concentration from the *S* measurement. Initially, we expected Co to be an n-type dopant and tried to replace it with Re. As expected, the *S* value of the sample with *x* = 0.2 showed n-type values (−10 μV K^−1^ at 300 K), but this changed to p-type with increasing *x* and its absolute value improved to 100 μV K^−1^ at 300 K for the sample with *x* = 0.5, which is close to that of the undoped sample (*x* = 0) [16]. These changes of *σ* and *S* suggest that a simple rigid-band approximation is not applicable to this system. As a result, we did not succeed in fabricating the desired n-type material with a high *S*.

The Co-substitution concentration dependence of *σ* and *S* is a rather complicated change and differs from those reported in previous studies on Ru [27] and Fe [28] substitution. Mizutani et al. reported an electron per atom ratio (*e*/*a*) for elements in the Periodic Table [29]: *e*/*a* = 1.31 for Re and *e*/*a* = 1.11 for Co. Using these *e*/*a* values of the constituent elements, the *e*/*a* value of *i*-Al–Pd–Re–Co QCs decreases with increasing Co concentration and, thus, *E*_F_ shifts to a lower energy, which seems to be an opposite way to obtain an n-type material. In contrast, in dilute substitution amounts of *x* = 0.1 and 0.2, Co appears to function as an n-type dopant. Hall coefficient measurements may clarify the underlying mechanism; however, the Hall voltage of *i*-Al–Pd–Re QC is too low to obtain reliable data at this stage.

**Figure 4:**
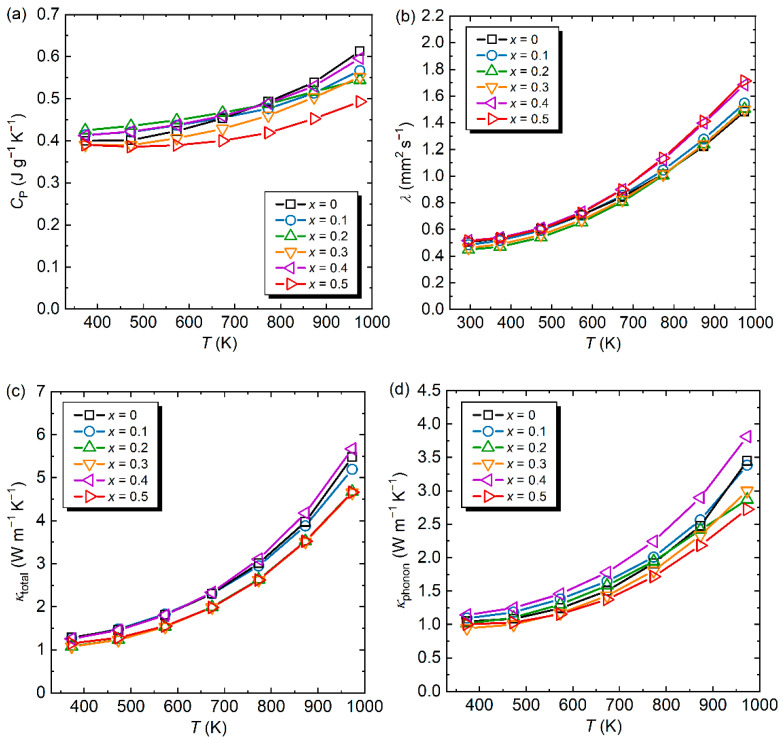
(**a**) Specific heat at constant pressure (*C*_P_), (**b**) thermal diffusivity (*λ*), (**c**) total thermal conductivity (*κ*_total_), and (**d**) phonon thermal conductivity (*κ*_phonon_) as functions of temperature for Al_71_Pd_20_(Re_1−*x*_Co*_x_*)_9_ (*x* = 0 [16], 0.1, 0.2, 0.3, 0.4, 0.5).

The temperature dependences of *C*_P_, *λ*, and *κ*_total_ from 373 to 973 K for all samples are shown in Figure 4a–c. The *C*_P_ values of the Al–Pd–Re–Co QCs were distributed between 0.39 and 0.41 J g^−1^ K^−1^ at 373 K and increased with the increasing temperature. The *λ* values at 373 K for all samples showed similar values of 0.40–0.42 mm^2^ s^−1^ and a temperature dependence. Table 2 lists *κ*_total_ at 373 K (*κ*_total,373K_) for all samples investigated. The *κ*_total,373K_ value decreased from 1.28 (*x* = 0) to 1.08 W m^−1^ K^−1^ (*x* = 0.3), probably due to the large decrease in *σ*, as shown in Figure 3a. Regarding the estimation of *κ*_electron_, the well-known Wiedemann–Franz law is not suited for QCs because it assumes that the spectral conductivity varies linearly with energy [30]. Maciá has discussed the validity of the Wiedemann–Franz law for QCs [31]. There is no empirical relationship to calculate *κ*_electron_ for QCs; thus, we adopted the conventional relationship shown in Equation (8) and *L*_0_ values using an empirical model (*L*_0_ = 1.5 + exp[−|*S*|/116] × 10^−8^ V^2^ K^−2^) by Kim et al. [32] for a rough estimation of *κ*_phonon:_

(8) $$\kappa_{\mathrm{phonon}}=\kappa_{\mathrm{total}}-L_{0}\sigma T.$$

The calculated *κ*_phonon_ as a function of temperature is shown in Figure 4d, and we list *κ*_phonon_ at 373 K (*κ*_phonon,373K_) for all samples in Table 2, together with the minimum thermal conductivity at 373 K (*κ*_min,373K_) using the Cahill model [33,34], which provides the lower limit of *κ*_phonon_ for amorphous solids and disordered crystals. The *κ*_min_ value can be calculated as follows:

(9) $$\kappa_{\min}={\left(\frac{\pi }{6}\right)}^{\frac{1}{3}}k_{B}n^{\frac{2}{3}}\sum_{\mathrm{long},\mathrm{trans}}v_{\mathrm{long},\mathrm{trans}}{\left(\frac{T}{\theta_{\mathrm{long},\mathrm{trans}}}\right)}^{2}\int_{0}^{\frac{\theta_{\mathrm{long},\mathrm{trans}}}{T}}\frac{x^{3}e^{x}}{{\left(e^{x}-1\right)}^{2}}dx.$$

Here, *k*_B_ is the Boltzmann constant, *n* is the number density of atoms, and *θ*_long,trans_ is the cut-off temperature, which is given by $\theta_{\mathrm{long},\mathrm{trans}}=v_{\mathrm{long},\mathrm{trans}}\left(\frac{\hbar }{k_{B}}\right){\left(6\pi^{2}n\right)}^{\frac{1}{3}}$, where ℏ is Planck’s constant. It should be noted that the apparent increase in *κ*_phonon_ at high temperatures, including for the Co-substituted samples, originates from conduction carriers [30]. The *κ*_phonon,373K_ was suppressed to 0.94 W m^−1^ K^−1^ by Co substitution, which is attributed to decreasing the phonon relaxation time (*τ*_phonon_):

(10) $$\kappa_{\mathrm{phonon}}=\frac{1}{3}C_{V}v_{s}^{2}\tau_{\mathrm{phonon}},$$

where *C*_v_ and *v*_s_ are the specific heat at constant volume and the effective speed of sound, respectively. Here, the *C*_V_ [35] and *v*_s_ can be expressed as:

(11) $$C_{V}=C_{P}-9VB\alpha^{2}T;$$

(12) $$v_{s}={\left[\frac{1}{3}\left(\frac{1}{v_{long}^{3}}+\frac{2}{v_{trans}^{3}}\right)\right]}^{-1/3},$$

where *V* and *α* are the atomic volume and the linear thermal expansion coefficient, respectively. Although we have no information on *α* for the present *i*-Al–Pd–Re–Co QCs, qualitative changes are discussed using *C*_P_.

Co-substitution for Re could reduce *κ*_phonon,373K_ from 1.05 (*x* = 0) to 0.94 (*x* = 0.3) W m^−1^ K^−1^, i.e., up to a 10% reduction. The *C*_P_ and *v*_s_ values did not change significantly between samples with *x* = 0 and 0.3 (less than ~2% for each) (Table 2), so *τ*_phonon_ is likely reduced because of the alloying effect. Here, we note that *κ*_phonon,373K_ of the sample with *x* = 0 is the same as the *κ*_min,373K_ value, and the observed *κ*_min,373K_ > *κ*_phonon,373K_ relationship is attributed to overestimation of *L*_0_ [36]. This trend was also observed in the Al–Pd–Re–Fe quaternary system [16].

The estimated elastic parameters of *ν*, *E*, *G*, and *B* for Al_71_Pd_20_(Re_1−*x*_Co*_x_*)_9_ (*x* = 0, 0.1, 0.2, 0.3, 0.4, 0.5) are listed in Table 3. The general mechanical properties of QCs are hard and brittle, so the estimated elastic moduli are consistent with the general trend. There is no literature on the mechanical properties of *i*-Al–Pd–Re QC. In general, an arc-melted and annealed *i*-Al–Pd–Re sample has a porous microstructure of a relative density of <70% because *i*-Al–Pd–Re QC forms from the peritectic reaction of Al_3_Pd_2_ and Al_11_Re_4_, and thus is very fragile [22]. We speculate that such a porous sample makes it difficult to measure intrinsic mechanical properties. The sintered bulk samples of *i*-Al–Pd–Re(–Co) QCs were dense enough to measure their intrinsic mechanical properties. Indeed, we obtained a higher *B* value of over 150 GPa for the sample with *x* = 0, which is close to Fe-based thermoelectric materials of Fe_3_Al_2_Si_3_, β-FeSi_2_, and FeAl_2_ [37]. It was found that Co substitution for Re slightly reduced the elastic moduli of *ν*, *E*, *G*, and *B*. The *ν* value is related to the volume change in the uniaxial deformation. Classical elastic theory predicts *ν* to be −1 to 0.5. A larger *ν* value means that a material possesses better plasticity. The estimated *ν* values of 0.260–0.303 for *i*-Al–Pd–Re–Co QCs are between those of ionic materials, such as NaCl (*ν* = 0.253) and CsCl (*ν* = 0.266) [38], and metallic materials, such as Aluminum 6061-T6 (*ν* = 0.33) and Cu (*ν* = 0.355) [38]. This may indicate that Co substitution for Re gives *i*-Al–Pd–Re QC a more ionic and brittle character. Compared with other *i*-QCs, such as *i*-Al–Pd–Mn QC [39,40] and *i*-Al–Cu–Fe QC [39], *i*-Al–Pd–Re–Co QCs had higher *ν* and *B* values, but the *G* value was almost identical. Decreasing *ν* will be associated with increasing covalency between atoms because covalent materials such as diamond and cubic boron nitride have low *ν* values of 0.069 and 0.14–0.18, respectively [38].

**Figure 5:**
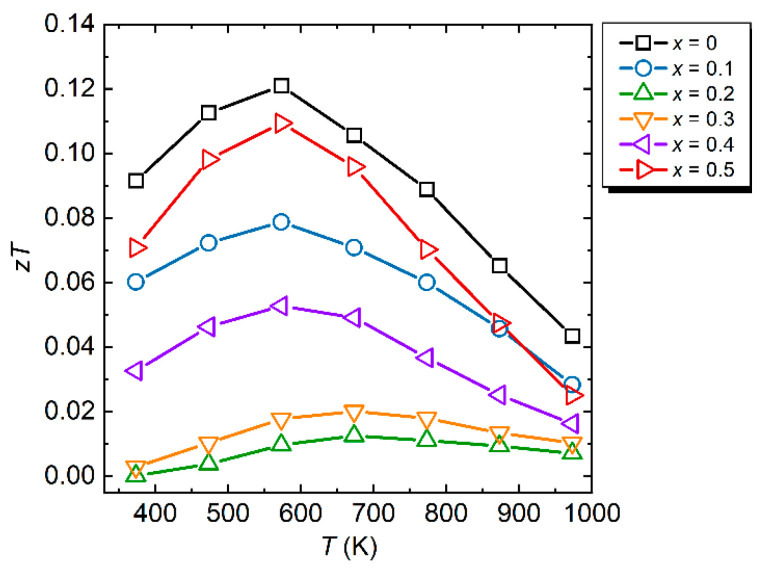
Dimensionless figure of merit (*zT*) as a function of temperature for Al_71_Pd_20_(Re_1−*x*_Co*_x_*)_9_ (*x* = 0 [16], 0.1, 0.2, 0.3, 0.4, 0.5).

Finally, we briefly mention *zT* as a function of temperature, as shown in Figure 5. The maximum *zT* value (*zT*_max_) was 0.12 at 573 K for the sample with *x* = 0. Although *κ*_total_ was reduced by the alloying effect via Co substitution for Re, *zT*_max_ was not enhanced because of the lowering of *S*^2^*σ* for all measured temperature ranges.

## 4. Conclusions

In this study, we systematically investigated the effect of Co substitution for Re for *i*-Al–Pd–Re QC on thermoelectric and mechanical properties. We found that the icosahedral quasi-crystalline phase can be synthesized by 50% replacement of Co for Re. Although *κ*_total_ was reduced by the alloying effect via Co substitution for Re, *zT*_max_ was not enhanced because of the lowering of *S*^2^*σ* for all measured temperature ranges. Co could produce n-type carriers in dilute substitution amounts of *x* = 0.1 and 0.2; however, *S* at 300 K showed an n- to p-type transition with increasing *x*. The observed effects of Co concentration on *σ* and *S* showed a complicated change, suggesting that a simple rigid-band approximation is not applicable to *i*-Al–Pd–Re QC. To develop a robust thermoelectric power generation module using Al-based QCs, we need to synthesize a high-performance n-type QC. This is a future challenge for the practical application of QCs.

## Figures and Tables

**Table 1:** Sintering temperature (*T*_S_), bulk densities (*d*_bulk_), calculated densities (*d*_calc_), and relative densities (*d*_bulk_*/d*_calc_) for Al_71_Pd_20_(Re_1−*x*_Co*_x_*)_9_ (*x* = 0 [16], 0.1, 0.2, 0.3, 0.4, 0.5).

Sample	*T*_S_ (K)	*d*_bulk_ (g cm^–3^)	*d*_calc_ (g cm^–3^)	*d*_bulk_*/d*_calc_ (%)
*x* = 0 [16]	1223	6.026	6.30 [22]	95.7
*x* = 0.1	1233	5.924	6.17	96.0
*x* = 0.2	1233	5.810	6.05	96.0
*x* = 0.3	1233	5.656	5.92	95.5
*x* = 0.4	1223	5.648	5.80	97.4
*x* = 0.5	1223	5.517	5.67	97.3

**Table 2:** Total thermal conductivity at 373 K (*κ*_total,373K_), phonon thermal conductivity at 373 K (*κ*_phonon,300K_), minimum thermal conductivity at 373 K (*κ*_min,373K_), longitudinal (*v*_long_) and transverse (*v*_trans_) speeds of sound, effective speed of sound (*v*_s_), rate of change in *v*_s_ (Δ*v*_s_/*v*_s_), specific heat at constant pressure at 373 K (*C*_P,373K_), and rate of change in *C*_P,373K_ (Δ*C*_P,373K_/*C*_P,373K_) for Al_71_Pd_20_(Re_1−*x*_Co*_x_*)_9_ (*x* = 0 [16], 0.1, 0.2, 0.3, 0.4, 0.5).

Sample	*κ*_total,373K_	*κ*_phonon,373K_	*κ*_min,373K_	*v*_long_/*v*_trans_
	(W m^–1^ K^–1^)	(W m^–1^ K^–1^)	(W m^–1^ K^–1^)	(m s^–1^)
*x* = 0 [16]	1.28	1.05	1.05	6430/3420
*x* = 0.1	1.27	1.09	1.07	6400/3500
*x* = 0.2	1.16	1.01	1.09	6430/3660
*x* = 0.3	1.08	0.94	1.06	6370/3490
*x* = 0.4	1.25	1.14	1.09	6480/3590
*x* = 0.5	1.15	1.00	1.11	6690/3660
Sample	*v*_s_	Δ*v*_s_/*v*_s_	*C*_P,373K_	Δ*C*_P,373K_/*C*_P,373K_
	(m s^–1^)	(%)	(J g^−1^ K^−1^)	(%)
*x* = 0 [16]	3820	-	0.3997	-
*x* = 0.1	3900	2.1	0.4138	3.5
*x* = 0.2	4070	6.5	0.4249	6.3
*x* = 0.3	3890	1.8	0.3902	−2.4
*x* = 0.4	4000	4.7	0.4124	3.1
*x* = 0.5	4080	6.8	0.3903	2.4

**Table 3:** Estimated elastic moduli (Poisson’s ratio (*ν*), Young’s modulus (*E*), shear modulus (*G*), and bulk modulus (*B*)) for Al_71_Pd_20_(Re_1−*x*_Co*_x_*)_9_ (*x* = 0, 0.1, 0.2, 0.3, 0.4, 0.5), and those of *i*-Al–Pd–Mn [39,40] and *i*-Al–Cu–Fe [39] QCs.

Sample	*ν*	*E*	*G*	*B*
	(-)	(GPa)	(GPa)	(GPa)
*x* = 0	0.303	184	70.5	155
*x* = 0.1	0.287	187	72.6	146
*x* = 0.2	0.260	196	77.8	136
*x* = 0.3	0.286	177	68.9	138
*x* = 0.4	0.279	186	72.8	140
*x* = 0.5	0.286	190	73.9	148
*i*-Al–Pd–Mn [39]	0.254	-	72.4	123
*i*-Al–Pd–Mn [40]	0.256	-	70.4	121
*i*-Al–Cu–Fe [39]	0.232	-	68.1	104

## Data Availability

The data presented in this study are available upon reasonable request from the corresponding author.
